# Application of Gurma Melon *(Citrullus lantus var. colocynthoides)* Pulp-Based Gel Fat Replacer in Mayonnaise

**DOI:** 10.3390/foods11182731

**Published:** 2022-09-06

**Authors:** Essam Mohamed Elsebaie, Mona Metwally Mousa, Samah Amin Abulmeaty, Heba Ali Yousef Shaat, Soher Abd-Elfttah Elmeslamy, Manal Salah Abbas Elgendy, Fatma M. Saleh, Rowida Younis Essa

**Affiliations:** 1 Food Technology Department, Faculty of Agriculture Kafrelsheikh University, Kafr El-Shiekh 33516, Egypt; 2 Food Science &Technology Department, Faculty of Home Economics, Al-Azhar University, Tanta 31512, Egypt; 3 Nutrition & Food Science Department, Faculty of Home Economics, Al-Azhar University, Tanta 31512, Egypt

**Keywords:** emulsion stability, GMPG, whiteness, viscosity, low fat

## Abstract

Gurma melon pulp-based gel (GMPG) was examined as a fat replacement in mayonnaise. GMPG was used to partially replace fat in quantities of 25, 50, and 70%, abbreviated as GMPG-25, GMPG-50, and GMPG-70, respectively. Mayonnaise’s physicochemical and sensory properties were studied. The data demonstrated that all low-fat mayonnaises had much lower energy value but significantly higher water content than their full-fat equivalents and that these differences developed as GMPG replacement levels increased. A microstructure analysis revealed compact the packing structures of big droplets in the whole fat sample and a baggy structure network of aggregated tiny droplets in the GMPG-25, GMPG-50, and GMPG-70 samples. There were no significant differences in pH or water activity after one day of storage between the full-fat and low-fat mayonnaises. Mayonnaises with GMPG-50 and GMPG-70 exhibited the same hardness as full-fat, whereas mayonnaises with GMPG-25 were harder than the other samples. Increased mayonnaise whiteness (L* increase and a* and b* reduction) was seen with reductions in fat. All samples had good sensory approval, with the 50% oil mayonnaise appearing to be the most appealing. It has been demonstrated that GMPG is an effective fat replacement agent.

## 1. Introduction

Mayonnaise has become one of the most widely consumed and commercialized sauces in the world due to its delicious flavor and attractive texture [1]. It is an oil/water emulsion in which egg proteins, particularly lipoproteins, function as emulsifiers. Steric forces have a major role in stabilizing mayonnaise emulsions [2]. Traditional mayonnaise has around 70–80% lipids, making it an extremely high-calorie and fat-dense food [3].

Customers are nevertheless concerned about its health effects, such as raising the risk of obesity and cardiovascular disease, despite the fact that it is manufactured from vegetable oils [4]. Due to the fact that fat is essential for such distinct product qualities, it might be difficult to create low-fat foods with similar texture and taste characteristics as full-fat foods [5].

Low-fat mayonnaise is created with 30–40% oil [6]. Many studies have demonstrated that fat replacements may be used to create mayonnaise that is low in fat [7]. For instance, polysaccharide gels that are high in dietary fiber are effective fat replacements [8]. More research is being conducted on polysaccharide gels, such as xanthan gums, guar gum, and pectin, as fat substitutes in food manufacturing, such as in low-fat meat products. However, there are not many articles that discuss mayonnaise with less fat. 

Gurma melon *(Citrullus lantus var. colocynthoides)* is a variety of watermelon that grows wild. It is a member of the *Cucurbitaceae* family [9]. Locally, it is known as the seed or Nubian melon. It has been farmed in Egypt since ancient times, maybe since the reign of the Pharaohs [10].

The economic significance of the Gurma melon has lately expanded since its production surpasses local consumption, allowing Egypt to export considerable amounts of its seeds. Furthermore, because of its resilience to drought and salt, it is an excellent crop for newly reclaimed fields [10]. It has been widely planted, particularly in Egypt’s northern area, due to its low water needs [11]. The seeds are the usable part of the fruit, while the remainder of the deseeded fruits are discarded into the environment, creating environmental issues. This wastage accounts for approximately 95.1% of the total fruit weight (65.35% pulp and 29.75% peel) [12]. These residues can be used as a source of some important vital compounds. Gurma melon pulp (GMP) contains a high percentage of pectin and proteins [12,13]. There are no studies about preparing Gurma melon pulp gel (GMPG) or using it as a substitute for fat in mayonnaise. Therefore, this study was conducted for the first time with the aim of using GMPG as a substitute for fat in mayonnaise in order to produce low-fat mayonnaise as well as to study the effects of this on the microstructure, physicochemical properties, viscosity, the stability of the emulsion, color, texture, and sensory properties of the resulting mayonnaise. 

## 2. Materials and Methods

### 2.1. Materials

Fifteen Gurma melon fruits (*Citrullus lanatus var. colocynthoides*) (about 45 kg) were collected from a local farm in Egypt’s El-Gharbia Governorate. Materials for the mayonnaise, including apple vinegar, eggs, salt, soybean oil, and sugar, were acquired from a local store. All of the chemicals were analytical-reagent-grade (extra-pure).

### 2.2. Methods

#### 2.2.1. Gurma Melon Pulp (GMP) Preparation 

The fruits were washed well with water. Then, the fruit was cut in half with a stainless-steel knife to remove the seeds. After the seeds were removed, each half was thoroughly washed with tap water to remove any remaining traces of seeds and their fibrous parts. After that, the outer green peel was removed. The pulp was divided into small pieces with a knife. It was dried at 50 °C until a constant weight was reached. The dried parts were crushed by a grinder (Moulinex AR1100, Pays de la Loire, France) then sieved through a 100-mesh sieve, and the resulting powder was packed into polyethylene bags and stored at 4 ± 1 °C until use.

#### 2.2.2. Gurma Melon Pulp Gel (GMPG) Preparation

GMPG was prepared by the method mentioned by Belluco et al. [14]. First, a citric acid solution was made by dissolving twenty-five grams of anhydrous citric acid in a liter of distilled water. GMP powder was added to the citric acid solution in a 1:10 (*w*/*v*) ratio. The resulting mixture was heated at 100 °C with continuous stirring for 20 min. Finally, the mixture was allowed to cool (until it reached room temperature) before being refrigerated at 4 ± 1 °C until used.

#### 2.2.3. Chemical Analysis of GMP Powder

The moisture, crude fat, ash, and crude protein amounts in GMP powder were measured using the methodologies recommended by AOAC [15]. Carbohydrates were calculated by subtracting the summation of the moisture, fat, ash, and protein percentages from 100. 

#### 2.2.4. GMPG Physical Property Measurement

The values of pH and water activity were determined at room temperature (twenty-five degrees Celsius). The pH was measured by a pH meter (HAANA HI902 m, Vöhringen, Germany), while the water activity was measured with a water activity meter (AQUALAB 4TE, Pullman, CA, USA). Each test was performed with six replicates for each sample. 

The color (L*, a*, and b*) was measured for each sample of GMP powder and each GMPG sample using a Hunter Lab colorimeter device (Colorflex, Broomfield, CO, USA). Estimates were performed in seven replicates for each sample.

**Hue angle = tan**^**−1**^b*/a* (1)

Chroma = [(b*)^**2**^+ (a*)^**2**^**]**^**1/2**^ (2)

#### 2.2.5. Mayonnaise Preparation

Mayonnaise samples were prepared using the method described by Worrasinchai et al. [16]. Mayonnaise samples (full-fat and low-fat) were prepared using the proportions shown for each ingredient in Table 1. In general, low-fat mayonnaise was prepared by replacing 25%, 50%, or 75% of the soybean oil with the same amount of GMPG. These percentages were determined based on the results of the preliminary experiments that were conducted.

In this study, three kilograms of each mayonnaise sample were made. In the beginning, vinegar and egg yolk were mixed in a plastic bowl, and the mixing process was carried out using a hand blender (moulinex DD1748EG, Turkey) at speed 2 for 30 s. Then, the rest of the other ingredients (including GMPG) were added, except the oil, and the mixture was blended again at speed 3 for a period of 90 s. At the end, the oil was added at a very slow rate (point by point), and the mixture was blended at speed 2 for two minutes after the completion of adding the required amount of oil. The resulting mayonnaise was poured into jars and stored at room temperature (25 ± 2 °C) until it was used in the analysis.

#### 2.2.6. Mayonnaise Composition Analysis

The moisture, ash, and protein percentages were estimated using the methods of AOAC [15]. The fat percentage was estimated using the approach of Marshall [17]. Carbohydrates were calculated by subtracting the summation of the moisture, fat, ash, and protein percentages from 100. 

#### 2.2.7. Caloric Value

Caloric values were computed using the equation of Liu et al. [1].

**Total calories = (4 × gm carbohydrate) + (9 × gm fat) + (4 × gm protein)** (3)

#### 2.2.8. Mayonnaise Microstructure

The microstructures of the mayonnaise samples under study were examined by placing a drop of each sample on a glass slide and examining it using a light microscope (Olympus cx-43, Olympus, Tokyo, Japan) to obtain micrographs at 100× magnification. 

#### 2.2.9. Mayonnaise Physicochemical Analyses

##### pH and Water Activity (Aw) 

Mayonnaise samples’ pH and *A*w values were measured as previously mentioned in Section 2.2.4. 

##### Bostwick Consistency Measurement

The Bostwick consistency test was performed using a Bostwick consistometer (CSC 24925000, CSC Scientificcompany, Richmond, VA, USA). In this test, the chamber of the device was filled with one hundred milliliters of the mayonnaise sample, and the distance that the sample moved was calculated after 30 s, starting from the time of releasing the gate of the device chamber directly. The test was performed with three measurements for each sample. 

##### Particle Size Measurement

The particle sizes of the mayonnaise samples were determined using the Mastersizer 3000 device (Malvern Panalytical Ltd., Worcestershire, UK). To determine the particle size of a sample, the mayonnaise sample (0.02 g) was diluted with 75 mL of a 1% sodium dodecyl sulfate solution. The sample’s relative index to soybean oil was adjusted to 1.460, and the absorbance was adjusted to 0.00. The D (4,3) diameter was used to obtain the average particle size [18].

##### Emulsion Stability

For the estimation of the emulsion stability of mayonnaise, a 20 g amount of the sample was weighted, put in centrifugation tubes, and spun for 30 min at 5000 rpm at room temperature (25 °C). The oil layer was evacuated, and the mass of the sediments was calculated. The next equation was used to calculate the stability of the mayonnaise emulsion [19].

(4) $$Thermal\;stability\left(\%\right)=\frac{Remainder\;emulsion\;weight}{Inatial\;emulsion\;weight}\times 100$$

##### Viscosity Measurement 

An HAAAKE Viscometer (model 7L, Grünheide, Germany) was used to measure the viscosity of mayonnaise at a shear rate of 10/S. After sample homogenization, all tests were completed with an L4 spindle at a regulated temperature of 25 ± 2 °C and a speed of 12 rpm, and the spindle was permitted to cycle five times for each sample. The viscosity of the sample was calculated by centipoise [20]. 

##### Mayonnaise Color Measurement 

Mayonnaise sample color parameter assessment was performed after a day of sample preparation by the approach previously outlined in Section 2.2.4.

ΔE = [(L*_sample_ − L*_control_)^2^ + (b*_sample_ − b*_control_)^2^ + (a*_sample_ − a*_control_)^2^]^1/2^ (5)

##### Texture Measurement 

The Stable Micro Systems Texture Analyzer TA-XT2 (Surrey, UK), with a 5 kg loading cell, was used to determine the textural parameters (hardness (N) and adhesiveness (N/S)) of mayonnaise. The mayonnaise was carefully poured into circular cylinders with inner and high diameters of 40 mm and 85 mm, respectively. A probing cylinder with a diameter of 10 mm was used to conduct penetration tests. One loop was completed to a sample depth of 10 mm at a constant crosshead velocity of 160 mm/min and then returned [21]. The measurement was performed in four replicates.

#### 2.2.10. Sensory Evaluation

The test was attended by twenty certified panelists from the Food Technology Department of Kafrelshiekh University’s Agriculture Faculty, ranging in age from 25 to 50 years. The panelists were selected based on their prior consumption of traditional mayonnaise. Furthermore, prior to testing, they were given a preliminary session in which each panelist extensively discussed and clarified each quality to be rated. The experiment was conducted in a portion of the laboratory that was lit with white fluorescent lights. The mayonnaise was kept at room temperature for one day after being made, as previously mentioned. The mayonnaise samples were placed in a covered dish with a three-digit code. The panelists evaluated the appearance, color (mayonnaise’s customary color attractiveness and creaminess), smell, taste, texture (uniformity and stiffness), and the total acceptability of the samples. Following each specimen’s examination, little flakes and water were used to freshen the taste. The sensory evaluation was carried out using a five-point hedonic scale ranging from 5 (like extremely) to 1 (dislike extremely) for each examined characteristic [16].

### 2.3. Statistical Analysis

A one-way analysis of variance (ANOVA) was used to analyze the data. Duncan’s multiple range test was used to differentiate the mean values of each group at a *p* ≤ 0.05 level.

## 3. Results and Discussion

### 3.1. Chemical Analysis of GMP Powder

Table 2 shows the findings of the GMP chemical composition analysis. The examination of the GMP powder revealed the following values: moisture 13.46%, fat (ether extracts) 2.14%, protein 11.38%, ash 3.85%, and total carbohydrates 69.17%. These findings, however, are generally in line with those of Abdelhady et al. [11], Salama et al. [12] and Korish [13].

According to a color study, the GMP powder was clear (L* = 90.73), only a little bit greenish (a* = −1.34), and slightly yellowish (b* = 22.61).

Similarly, fat replacer made with the GMP powder was also clear (L* = 92.85), somewhat greenish (a* = −1.59), and yellowish (b* = 18.37) (Table 2).

Due to the presence of citric acid during gelification, the fat replacement had an acidic pH (3.49). Because GMP powder contains a lot of pectin, acidification is crucial. pH decreases cause a reduction in the dissociation of carboxylic groups, which increases the contact areas between molecules and water retention and creates a gel [22]. The fat substitute has a high water activity value (0.94), which translates to a lot of free water (Table 2).

### 3.2. Chemical Composition and Caloric Values

Table 3 presents the approximate composition and calorie values of full-fat (control) and low-fat mayonnaises including GMPG. Due to GMPG’s extremely high moisture content, a common property of carbohydrate-based fat replacers, the moisture content rose as the degree of GMPG addition increased [23]. Low-fat samples had substantially lower fat contents than full-fat samples due to the difference in oil content added to the full-fat and low-fat models. In terms of ash and protein composition, there was no discernible difference between the full-fat and low-fat mayonnaises. The carbohydrate contents marginally rose as a function of increasing the percentage of fat replacement.

The caloric value was reduced by replacing the oil with GMPG since lipids, which have a caloric value of 9 kcal/g and are more calorically dense than carbs and proteins (4 kcal/g), account for the majority of the caloric value. The low-fat mayonnaise formulations’ calorie values were significantly (*p* ≤ 0.05) decreased by increasing the GMPG replacer levels. The pectin, which makes up the majority of the GMPG sample in addition to water, has no calories itself since it is neither absorbed nor digested by the human gastrointestinal system [1].

### 3.3. Mayonnaise Microstructure

**Figure 1:**
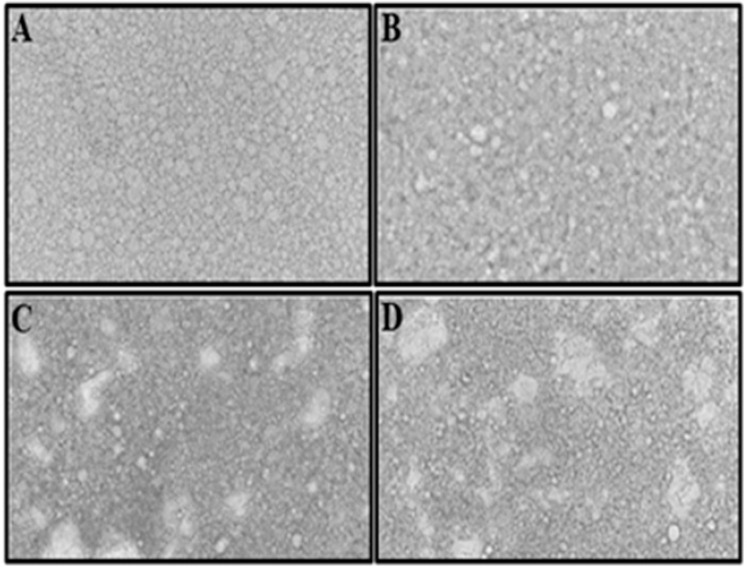
Optical microscope graphs of full-fat mayonnaise (**A**) and mayonnaise with (**B**) 25%, (**C**) 50%, and (**D**) 70% substitution of oil with Gurma melon pulp gel (GMPG).

The microstructures of the various mayonnaises, which comprise oil droplets scattered in a water phase, were evaluated using optical microscopy [24]. Figure 1 is illustrative and shows the differences between the full-fat and low-fat mayonnaise microstructure photographs. Full-fat mayonnaise has an oil content exceeding 75%, so the traditional emulsion microstructure of sphere droplets stacked inside a continuous phase transition was not seen. Instead, oil droplets abandoned their sphericity and assumed a deformed shape that preferred close stacking and the contact of fat globules, giving mayonnaise its distinctive consistency [25]. The microstructure of full-fat mayonnaise was consistent with expectations, showing significant interactions between tiny and big fat droplets that were tightly packed together (Figure 1A). Droplet agglomeration and void-interspaced areas increased when the fat level was reduced in low-fat mayonnaises (Figure 1B,D). The agglomeration and void-interspaced areas in the low-fat mayonnaise images may be due to the GMPG’s thickening ability and the emulsion’s continuous phase gelling phenomena.

### 3.4. Mayonnaise Physicochemical Properties

All prepared mayonnaises have a well-formed emulsion with an appropriate consistency.

#### 3.4.1. Mayonnaise pH and Water Activity (Aw) 

Table 4 shows the pH, water activity (Aw), and running distance results for full-fat and low-fat mayonnaises after one day of room temperature storage. After one-day of storage, there was no significant (*p* ≤ 0.05) variation in the pH values (3.96–3.78) of all formulations. 

Nevertheless, introducing GMPG as a fat replacer decreased the pH values in mayonnaise recipes, most likely due to GMPG’s strong acidity (pH = 3.49, Table 2). According to basic guidelines for microbiological safety, mayonnaise produced with unpasteurized eggs should be made with vinegar with a pH of 4.1 or below and kept at room temperature (18 to 22 °C) for a minimum of 24 h to limit the danger of bacteria [26]. The Aw of mayonnaises rose, as predicted, as the amount of GMPG replacement increased, owing to the increased water content of the recipes. All the mayonnaises that were evaluated had high Aw values, ranging from 0.948 to 0.988. 

According to Chirife et al. [27], the Aw of full-fat mayonnaises (77 to 79% oil) was around 0.93, whereas that of low-fat samples (37 to 41% oil) was higher, around 0.95. The high levels of Aw in the products make pH and heat treatment essential to ensure the product’s microbial safety. 

#### 3.4.2. Mayonnaise Consistency 

The consistency of mayonnaise was evaluated by a Bostwick consistometer. This simple and quick analytical equipment is frequently employed by food producers to evaluate the quality and standard conformity of their products. A short running distance suggests a high mayonnaise consistency, which is typical of full-fat mayonnaise containing 80% fat and having a thick and heavy body structure [28]. The short running distance that all samples displayed (Table 4) indicated strong product consistency. Only GMPG-70 (a formulation with a 70% substitution of oil with Gurma melon pulp gel) showed a marginally but considerably longer running distance compared to the other samples. The lower fat and higher water levels in the product were responsible for the lowest Bostwick consistency values of the GMPG-70 sample.

#### 3.4.3. Particle Size Analysis

Table 4 displays the difference in droplet size between full-fat and reduced-fat mayonnaise emulsions. The mayonnaise emulsions with GMPG added had an average droplet size of 1.95- 4.21 µm. Full-fat samples had the largest oil droplet size (9.38 µm) when compared to low-fat samples. The more GMPG added, the smaller and denser the emulsion droplets became. GMPG-25 (formulation with 25% replacement of oil with Gurma melon pulp gel) had the biggest particle size (4.21 µm), and GMPG-70 (formulation with 70% substitution of oil with Gurma melon pulp gel) had the lowest droplet size (1.95 µm) among the reduced-fat mayonnaises. This observation matched the results from Liu et al. [1]. Furthermore, oil droplet size is simply one measure of viscosity and emulsion stabilization [27].

#### 3.4.4. Emulsion Stability

The stability of full-fat and reduced-fat mayonnaises was tested using a technique previously published by Mun et al. [29] to evaluate the emulsion stability of mayonnaise. All of the mayonnaises created in this investigation were highly stable (>99%), with no water or oil loss (Table 4). As a result of the aqueous layer viscosity increase caused by the addition of GMPG, which decelerated oil droplet movement, the low-fat mayonnaise samples demonstrated greater stability than the full-fat samples [30]. As a result of these findings, the GMPG might be utilized to make low-fat mayonnaise as a stabilizing fat substitute.

The creaming, flocculation, and coalescence of droplets are frequently linked to emulsion instability. Because of their close proximity, oil droplets are condensed and unable to flow freely in high-fat products such as full-fat mayonnaise. The motion of the oil droplets in low-fat products is typically decreased with the addition of thickening ingredients such as starches or gums in the water phase [31]. The enhanced stiffness structure of the fat replacer gel in the low-fat mayonnaise used in this study may have mimicked the technical functionality of the aforementioned additives, producing powerful gel-like interactions.

#### 3.4.5. Viscosity Measurement

Mayonnaise exhibits pseudoplastic behavior since it is a non-Newtonian fluid. As a result, apparent viscosity was utilized rather than viscosity. According to Table 4, the full-fat mayonnaise sample had a viscosity of 17.42 cP, but the viscosity values of the reduced-fat mayonnaise treatments made by adding GMPG ranged between 15.89 and 21.13 cP. The formulation with GMPG-25 (a sample that substitutes 25% of the oil with Gurma melon pulp gel) had the maximum viscosity value. According to Worrasinchai et al. [16], the reason why the GMPG-25 sample had the maximum viscosity was because of the GMPG’s gelling/thickening impact and the establishment of a robust three-dimensional structured network between the oil droplets and the fat replacer. Additionally, it was noted from the data that the apparent viscosity was reduced with an increase in the rate of GMPG addition. This result may be explained by the product’s higher water and lower fat contents.

#### 3.4.6. Mayonnaise Color

The color characteristics of the full-fat and reduced-fat mayonnaises are shown in Table 5. Color, particularly lightness (L*), is a significant feature of mayonnaise’s appearance and, as a result, acceptance [29]. Full-fat mayonnaise had the smallest L* value (82.35) when compared to its reduced-fat equivalent (between 85.27 and 88.26). According to Chantrapornchai et al. [32], the emulsion transformed from a grey to an increasingly dazzling white color as the droplet size decreased due to increased light scattering. Because of the substantially smaller droplet size, the L value of GMPG-70 (a formulation with a 70% substitution of oil with Gurma melon pulp gel) was much greater than that of full-fat or any other low-fat samples. Low-fat mayonnaises had significantly lower a* (redness) and b* (yellowness) values compared to their full-fat counterparts. Furthermore, data from the same table demonstrated that when the levels of GMPG substitution increased, the a values (redness) and b values (yellowness) declined.

The hue angle values of GMPG-25 (a formulation with a 25% substitution of oil with Gurma melon pulp gel) and GMPG-50 (a formulation with a 50% substitution of oil with Gurma melon pulp gel) were higher than in the other samples due to the higher a* values. The hue angle values of all samples ranged from 97.78 to 99.40, indicating that the mayonnaise samples were yellowish in color.

The model low-fat mayonnaises made with GMPG demonstrated the lowest total color differences (ΔE) irrespective of the GMPG concentration.

#### 3.4.7. Mayonnaise Texture

**Figure 2:**
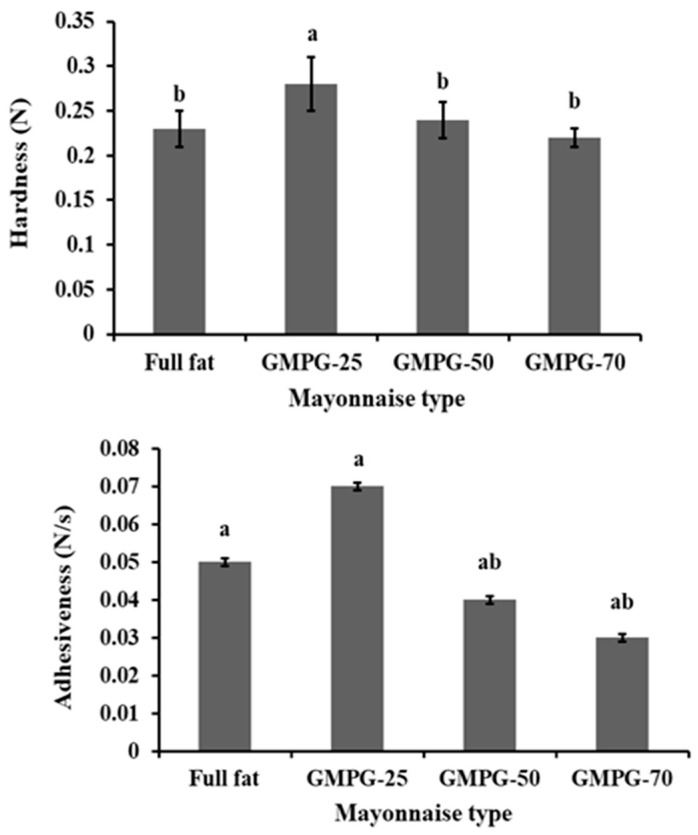
Hardness and adhesiveness of full-fat mayonnaises and mayonnaise with replacement of oil by Gurma melon pulp gel (GMPG). GMPG-25, GMPG-50, and GMPG-70: formulations with 25%, 50%, and 70% substitutions of oil with Gurma melon pulp gel (GMPG). Data are presented as means ± SD; Values followed by the same letter in each row are not significantly different at *p* ≤ 0.05.

Throughout the texture study, mayonnaise hardness and adhesiveness were assessed in particular since they are essential textural properties impacting the workability and mouthfeel of the product and are connected to the flow and viscosity behavior of the system [33]. The hardness of all reduced-fat mayonnaises was greater than or equivalent to their full-fat competitors (Figure 2). GMPG-25, for instance, was substantially harder (0.28 N) than full-fat (0.23 N), GMPG-50 (0.24 N), and GMPG-70 (0.22 N), which were all comparable. It is possible to infer that the fat replacer employed in the creation of reduced-fat mayonnaises was capable of providing structural strength to the product despite the large fat reduction obtained. GMPG-25 was likewise shown to be much stickier than all other formulations, although GMPG-50 and GMPG-70 were less adhesive than the full-fat competitor. 

The maximum hardness and adhesiveness of the GMPG-25 mayonnaise compared to all other samples (including the full-fat equivalent) may be attributed to an increase in the viscosity of the continuous phase owing to the gelling/thickening action of GMPG and the production of a robust tri-dimensional structured network between oil droplets and the fat replacer, as reported by Worrasinchai et al. [16]. The structured network was weaker in GMPG-70 and GMPG-50 than in GMPG-25, most likely due to less oil in the dispersed layer. 

### 3.5. Sensory Evaluation

**Figure 3:**
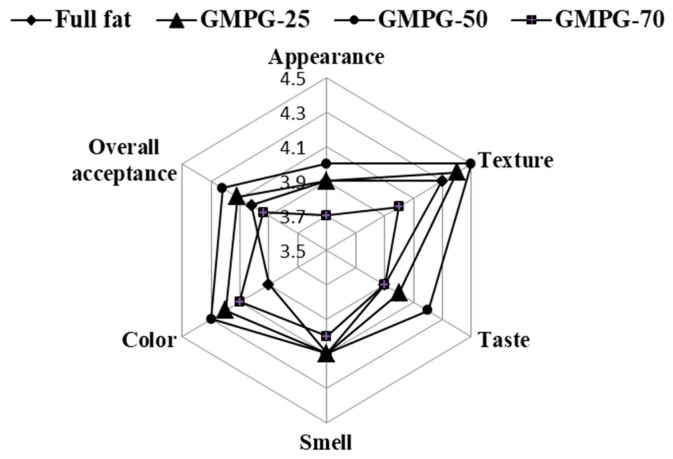
Sensory evaluation of full-fat mayonnaises and mayonnaise with replacement of oil by Gurma melon pulp gel (GMPG). GMPG-25, GMPG-50, and GMPG-70: formulations with 25%, 50%, and 70% substitutions of oil with Gurma melon pulp gel (GMPG).

The sensory assessment scores of full-fat and low-fat mayonnaises are presented in Figure 3. The GMPG-25 and GMPG-50 samples showed no statistical difference in appearance, texture, and smell scores from the full-fat one, whereas the GMPG-70 sample gave lower scores for these attributes. Full-fat samples gave lower scores for color and taste attributes; meanwhile, the GMPG-25 and GMPG-50 samples gave the highest scores.

Overall sensory acceptance was favorable for all samples, with the GMPG-50 and GMPG-25 mayonnaise being considerably favored by a consumer panel, followed by full-fat and GMPG-70. GMPG-50 received the highest overall score. Full-fat and GMPG-70 had the lowest overall score but a rating approaching 4 (somewhat like) for consistency. The excellent findings achieved, particularly the highest grades on GMPG-50, are encouraging, especially given that no information about the product’s health claim was supplied to the judges throughout the test. Our results were in accordance with Liu et al. [1] and Worrasinchai et al. [16]. 

## 4. Conclusions

Gurma melon pulp-based gel has been effectively employed as a fat substitute in the manufacture of low-fat mayonnaise. According to the findings of this study, GMPG served a dual role as a fat replacer and an emulsion stabilizer, resulting in low-fat, low-calorie mayonnaises. The reduced-fat mayonnaise produced had a Bostwick consistency, texture, viscosity qualities, and stability comparable to or greater than the full-fat equivalent, demonstrating the capacity of the GMPG to compensate for the lack of fat in the clean-label items. Oil replacement levels of up to 70% were acceptable. Nonetheless, the optimal substitute levels were determined to be 50% of the utilized oil.

## Figures and Tables

**Table 1:** Mayonnaise recipes.

Ingredients	Mayonnaise Formulation (% wt)			
	Full-Fat	GMPG-25	GMPG-50	GMPG-70
**soybean oil**	82.20	61.65	41.10	24.67
**fat replacer**	-	20.55	41.10	57.53
**egg yolk**	7.27	7.27	7.27	7.27
**vinegar (5%)**	8.72	8.72	8.72	8.72
**salt**	0.73	0.73	0.73	0.73
**mustard**	0.36	0.36	0.36	0.36
**sugar**	0.36	0.36	0.36	0.36
**ground white pepper**	0.35	0.36	0.36	0.36

GMPG-25, GMPG-50, and GMPG-70: formulations with 25%, 50% and 70% substitutions of oil with Gurma melon pulp gel (GMPG).

**Table 2:** Approximate chemical composition and color parameters (L*, a*, and b*) evaluated in Gurma melon pulp powder and color parameters (L*, a*, and b*), pH, and water activity (Aw) evaluated in fat replacer based on Gurma melon pulp powder.

Parameters	GMP Powder
**Moisture (%)**	13.46 ± 0.32
**Fat (%)**	2.14 ± 0.09
**Proteins (%)**	11.38 ± 0.55
**Ash (%)**	3.85 ± 0.19
**Total Carbohydrates (%)**	69.17 ± 0.71
L*	90.73 ± 2.07
a*	−1.34 ± 0.19
b*	22.61 ± 0.40
**Hue angle**	93.39 ± 1.13
**Chroma**	22.65 ± 0.52
**GMPG**	
L*	92.85 ± 2.88
a*	−1.59 ± 0.12
b*	18.37 ± 1.03
**Hue angle**	94.95 ± 1.39
**Chroma**	18.44 ± 0.63
**pH**	3.49 ± 0.01
**water activity (Aw)**	0.94 ± 0.002

GMP: Gurma melon pulp, GMPG: Gurma melon pulp gel.

**Table 3:** Proximate analysis (%, *w*/*w*) and caloric values of mayonnaise samples at different fat contents.

Parameters	Mayonnaise Formulation			
	Full-Fat	GMPG-25	GMPG-50	GMPG-70
**Moisture (%)**	12.30 ± 0.09 ^d^	33.27 ± 0.49 ^c^	51.36 ± 0.26 ^b^	66.25 ± 0.17 ^a^
**Fat (%)**	85.02 ± 0.48 ^a^	62.86 ± 1.21 ^b^	43.66 ± 1.60 ^c^	27.94 ± 0.12 ^d^
**Proteins (%)**	1.25 ± 0.06 ^a^	1.43 ± 0.10 ^a^	1.60 ± 0.08 ^a^	1.73 ± 0.10 ^a^
**Ash (%)**	0.85 ± 0.02 ^a^	0.89 ± 0.02 ^a^	0.94 ± 0.01 ^a^	0.97 ± 0.02 ^a^
**Total Carbohydrates (%)**	0.58 ± 0.01 ^c^	1.55 ± 0.23 ^b^	2.44 ± 0.38 ^ab^	3.11 ± 0.45 ^a^
**Caloric values (kcal/100 g)**	772.50 ± 2.10 ^a^	577.66 ± 4.65 ^b^	409.1 ± 5.82 ^c^	270.82 ± 1.94 ^d^

GMPG-25, GMPG-50, and GMPG-70: formulations with 25%, 50%, and 70% substitutions of oil with Gurma melon pulp gel (GMPG). Data are presented as means ± SD; Assays were performed in triplicate; Total carbohydrates were calculated by difference; Values followed by the same letter in each row are not significantly different at *p* ≤ 0.05.

**Table 4:** pH, water activity (aw), running distance, particle size, viscosity, and emulsion stability of full-fat mayonnaises and mayonnaise with the replacement of oil by Gurma melon pulp gel (GMPG).

Parameters	Mayonnaise Type			
	Full-Fat	GMPG-25	GMPG-50	GMPG-70
**pH**	3.96 ± 0.09 ^a^	3.85 ± 0.10 ^a^	3.81 ± 0.06 ^a^	3.78 ± 0.09 ^a^
**Water activity (Aw)**	0.948 ± 0.004 ^a^	0.979 ± 0.003 ^a^	0.985 ± 0.002 ^a^	0.988 ± 0.003 ^a^
**Running distance (cm)**	0.2 ± 0.1 ^b^	0.1 ± 0.00 ^b^	0.3 ± 0.1 ^b^	0.5 ± 0.1 ^a^
**Particle size (µm)**	9.38 ± 0.12 ^a^	4.21 ± 0.05 ^b^	2.37 ± 0.05 ^c^	1.95 ± 0.07 ^d^
**Stability**	99.8 ± 0.43 ^b^	99.8 ± 0.72 ^b^	100 ± 0.54 ^a^	99.7 ± 0.39 ^b^
**Viscosity (cP)**	17.42 ± 1.1 ^b^	21.13 ± 0.97 ^a^	16.60 ± 0.85 ^b^	15.89 ± 0.94 ^bc^

GMPG-25, GMPG-50, and GMPG-70: formulations with 25%, 50%, and 70% substitutions of oil with Gurma melon pulp gel (GMPG). Data are presented as means ± SD; Each value was an average of three replicates; Values followed by the same letter in each row are not significantly different at *p* ≤ 0.05.

**Table 5:** Color parameters (L*, a*, and b*) of full-fat mayonnaises and mayonnaise with replacement of oil by Gurma melon pulp gel (GMPG).

Parameters	Mayonnaise Type			
	Full-Fat	GMPG-25	GMPG-50	GMPG-70
L*	82.35 ± 0.87 ^c^	85.27 ± 0.82 ^b^	87.62 ± 0.94 ^a^	88.26 ± 0.93 ^a^
a*	−3.19 ± 0.28 ^c^	−3.42 ± 0.26 ^ab^	−3.62 ± 0.30 ^a^	−3.39 ± 0.22 ^b^
b*	23.36 ± 0.94 ^ab^	22.90 ± 1.02 ^b^	21.86 ± 0.95 ^bc^	22.88 ± 0.97 ^b^
**Hue angle**	97.78	98.49	99.40	98.43
**Chroma**	23.58	23.15	22.16	23.13
ΔE	0	2.96	5.50	5.93

GMPG-25, GMPG-50, and GMPG-70: formulations with 25%, 50%, and 70% substitutions of oil with Gurma melon pulp gel (GMPG). Data are presented as means ± SD; Each value was an average of three replicates; Values followed by the same letter in each row are not significantly different at *p* ≤ 0.05.

## Data Availability

The authors confirm that the data supporting the findings of this study are available within the article.

## References

[1] Liu H., Xu X., Guo S.D. (2007). Rheological, texture and sensory properties of low-fat mayonnaise with different fat mimetics. LWT-Food Sci. Technol..

[2] Gaurav G., Rathna K., Ken C., Bruce C. (2010). Emulsifying functionality of enzyme-modified milk proteins in O/W and mayonnaise-like emulsions. Afr. J. Food Sci..

[3] Honold P.J., Jacobsen C., Jónsdóttir R., Kristinsson H.G., Hermund D.B. (2016). Potential seaweed-based food ingredients to inhibit lipid oxidation in fish-oil-enriched mayonnaise. Eur. Food Res. Technol..

[4] Evanuarini H., Hastuti P. (2015). Characteristic of low fat mayonnaise containing porang flour as stabilizer. Pak. J. Nutr..

[5] Ali T.M., Waqar S., Ali S., Mehbsoob S., Hasnain A. (2015). Comparison of textural and sensory characteristics of low-fat mayonnaise prepared from octenyl succinic anhydride modified corn and white sorghum starches. Starch-Stärke.

[6] Lee I., Lee S., Lee N., Ko S. (2013). Reduced-fat mayonnaise formulated with gelatinized rice starch and xanthan gum. Cereal Chem..

[7] Rahmati N.F., Mazaheri Tehrani M., Daneshvar K., Koocheki A. (2015). Influence of selected gums and pregelatinized corn starch on reduced fat mayonnaise: Modeling of properties by central composite design. Food Biophys..

[8] Laneuville S.I., Paquin P., Turgeon S.L. (2005). Formula optimization of a low-fat food system containing whey protein isolate-xanthan gum complexes as fat replacer. J. Food Sci..

[9] Ziyada A., Elhussien S. (2008). Physical and ChemicalCharacteristics of *Citrullus lanatus* Var. Colocynthoide Seed Oil. J. Phys. Sci..

[10] El-Shabrawy R., Hatem A. (2008). Effect of sowing date and plant distribution system on growth and yield of gurma watermelon (*Citrullus lanatus* var. colocynthoides). J. Plant Prod..

[11] Abdelhady M., Masoud M., Elbaz S. (2014). Production of bioethanol from Gurma watermelon wastes. J. Biol. Chem. Environ. Sci.

[12] Salama I., Abo-Elmaaty S., Sulieman A., Abdel-Hady M. (2019). Innovation of Jam from Gurma Melon Pulp as Un Traditional Source. Zagazig J. Agric. Res..

[13] Korish M. (2015). Potential utilization of *Citrullus lanatus* var. Colocynthoides waste as a novel source of pectin. J. Food Sci. Technol..

[14] Belluco C.Z., Mendonça F.J., Zago I.C.C., Di Santis G.W., Marchi D.F., Soares A.L. (2022). Application of orange albedo fat replacer in chicken mortadella. J. Food Sci. Technol..

[15] Horwitz W., Latimer G., AOAC (Association of Official Agricultural Chemists) (2016). The Official Methods of Analysis of AOAC International.

[16] Worrasinchai S., Suphantharika M., Pinjai S., Jamnong P. (2006). β-Glucan prepared from spent brewer’s yeast as a fat replacer in mayonnaise. Food Hydrocoll..

[17] Marshall R.T. (1992). Standard Methods for the Examination of Dairy Products.

[18] Roland I., Piel G., Delattre L., Evrard B. (2003). Systematic characterization of oil-in-water emulsions for formulation design. Int. J. Pharm..

[19] Karshenas M., Goli M., Zamindar N. (2018). The effect of replacing egg yolk with sesame–peanut defatted meal milk on the physicochemical, colorimetry, and rheological properties of low-cholesterol mayonnaise. Food Sci. Nutr..

[20] Bourne M. (2002). Food Texture and Viscosity: Concept and Measurement.

[21] Nikzade V., Tehrani M.M., Saadatmand-Tarzjan M. (2012). Optimization of low-cholesterol–low-fat mayonnaise formulation: Effect of using soy milk and some stabilizer by a mixture design approach. Food Hydrocoll..

[22] Damodaran S., Parkin K.L. (2019). IQuímica de Alimentos de Fennema.

[23] Akoh C.C. (1998). Fat replacers. Food Technol..

[24] Langton M., Jordansson E., Altskär A., Sørensen C., Hermansson A.-M. (1999). Microstructure and image analysis of mayonnaises. Food Hydrocoll..

[25] Depree J., Savage G. (2001). Physical and flavour stability of mayonnaise. Trends Food Sci. Technol..

[26] Jay J.M., Loessner M.J., Golden D.A. (2008). Modern Food Microbiology.

[27] Chirife J., Vigo M.S., Gomez R.G., Favetto G.J. (1989). Water activity and chemical composition of mayonnaises. J. Food Sci..

[28] Duncan S.E., Smith J.S., Hui Y.H. (2014). Fats: Mayonnaise. Food Processing: Principles and Applications.

[29] Mun S., Kim Y.-L., Kang C.-G., Park K.-H., Shim J.-Y., Kim Y.-R. (2009). Development of reduced-fat mayonnaise using 4αGTase-modified rice starch and xanthan gum. Int. J. Biol. Macromol..

[30] Manoj P., Fillery-Travis A.J., Watson A.D., Hibberd D.J., Robins M.M. (2000). Characterization of a polydisperse depletion-flocculated emulsion: III. Oscillatory rheological measurements. J. Colloid Interface Sci..

[31] Mcclements D.J. (2007). Critical review of techniques and methodologies for characterization of emulsion stability. Crit. Rev. Food Sci. Nutr..

[32] Chantrapornchai W., Clydesdale F., McClements D.J. (1999). Influence of droplet characteristics on the optical properties of colored oil-in-water emulsions. Colloids Surf. A Physicochem. Eng. Asp..

[33] Chang C., Li J., Li X., Wang C., Zhou B., Su Y., Yang Y. (2017). Effect of protein microparticle and pectin on properties of light mayonnaise. LWT-Food Sci. Technol..

